# Ectopic Cushing’s syndrome due to a mesenteric neuroendocrine tumour

**DOI:** 10.1308/003588412X13373405387492

**Published:** 2012-05

**Authors:** N Mashoori, AH Rabani, AR Kazemeini

**Affiliations:** Imam Khomeini Hospital, Tehran University of Medical Sciences, Tehran,Iran

**Keywords:** Ectopic Cushing’s syndrome, Mesenteric, Neuroendocrine tumour

## Abstract

**INTRODUCTION:**

Neuroendocrine tumours (NETs) are tumours that commonly involve the gastrointestinal system. Common primary sites in the gastrointestinal system include the small intestine, appendix, rectum and pancreas. Mesenteric NETs are extremely rare entities and are sparsely reported in the literature.

**CASE HISTORY:**

We report the case of a 62-year-old woman with ectopic Cushing’s syndrome due to excessive adrenocorticotropic hormone secretion by a primary mesenteric tumour in the small intestine and its liver metastases.

**CONCLUSIONS:**

Although rare, the mesentery can be a primary site for NETs. It can cause similar symptoms and require similar treatment options. Tumour resection and debulking are acceptable ways to improve both the survival and symptoms.

Neuroendocrine tumours (NETs) originate from neuroendocrine cells, which are widely distributed throughout the body. They secret different substances such as somatostatin, gastrin and adrenocorticotropic hormone (ACTH). Excess amounts of these substances can lead to various clinical presentations depending on the substances produced by the tumour. NETs most commonly involve the lungs and gastrointestinal system. They have also been occasionally reported in ovaries, the prostate, lymph nodes and the cervix. Common primary sites in the gastrointestinal system include the small intestine, appendix, rectum and pancreas. Mesenteric NETs are extremely rare and are often secondary to another primary tumour located elsewhere.

We report the case of a 62-year-old woman with ectopic Cushing’s syndrome that was caused by excessive ACTH production by a tumour located in the small intestinal (ileum) mesentery and its liver metastases.

## Case history

Our patient was admitted to the endocrinology ward due to new onset diabetes, concomitant hypertension and cushingoid appearance. She had a six-year history of rheumatoid arthritis and was being treated with prednisolone. She reported a history of a benign thyroid nodule that had been biopsied 20 years previously. She also had a history of resolved peptic ulcer disease. She was suspected primarily to have exogenous Cushing’s syndrome and her prednisolone was therefore discontinued. Serum ACTH and 24-hour urine cortisol were measured and both were within the normal range. Because the serum ACTH level was not suppressed in response to a high dose dexamethasone suppression test, Cushing’s syndrome due to ectopic ACTH secretion was considered as the diagnosis.

**Figure 1:**
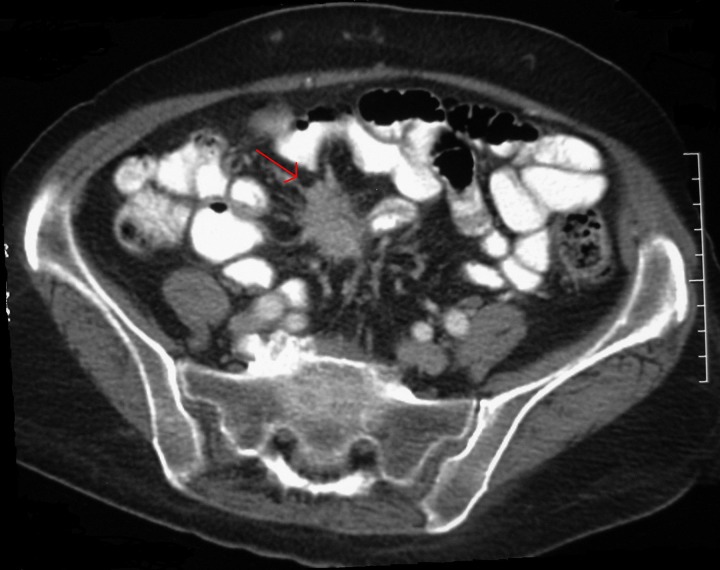
Mesenteric mass

**Figure 2:**
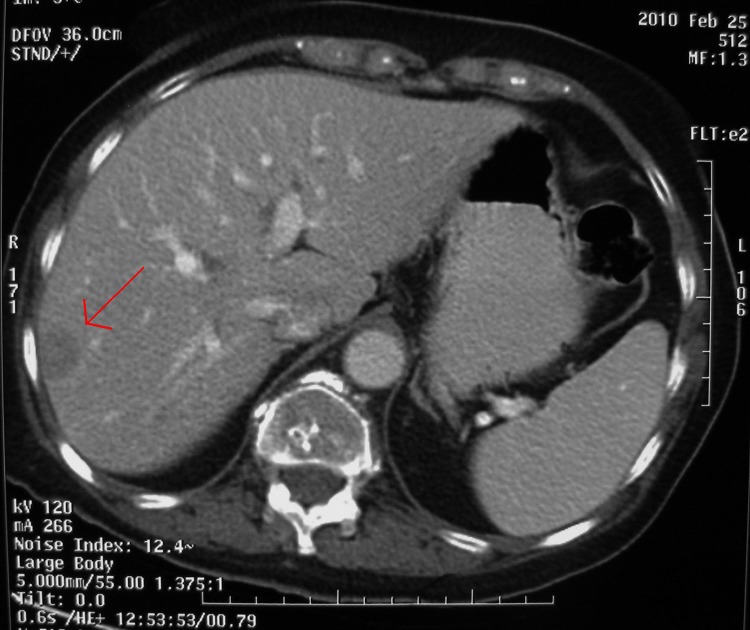
Liver metastases

In order to localise the source of ACTH secretion, the patient had an octreotide scan and computed tomography (CT) of the chest, abdomen and pelvis. The abdominal CT demonstrated a mass in the small bowel mesentery with speculated margins and some hepatic lesions in the right lobe suggestive of metastasis (Figs 1 and 2). In the octreotide scan, increased absorption in the abdomen was consistent with the location of the mesenteric mass and hepatic lesions. The patient underwent a percutaneous liver biopsy. Pathological examination of the tissue was consistent with the diagnosis of NET. With this new diagnosis, her symptoms were reviewed. She had no history of abdominal pain, changes in bowel habits or rectal bleeding; neither did she have any complaints of flushing or cardiopulmonary symptoms. Serum 5-hydroxyindoleacetic acid and vanillylmandelic acid were within normal limits.

To exclude other possible primary sites, the patient underwent a small bowel series and colonoscopy. Neither revealed any pathological findings. Meanwhile, she was transferred to the intensive care unit because of decreased consciousness due to uncontrolled hypertension and diabetes mellitus. Owing to being high risk for surgery at the time, she was not scheduled for surgical intervention. She underwent monthly injection of octreotide acetate. Chemoembolisation was performed for her metastatic lesions in the liver while she was preparing for a surgical intervention. As her symptoms could not be controlled with medical treatment, she was scheduled for surgical intervention (mesenteric mass resection, resection of liver metastases if possible and bilateral adrenalectomy for symptomatic control) one year following diagnosis.

**Figure 3:**
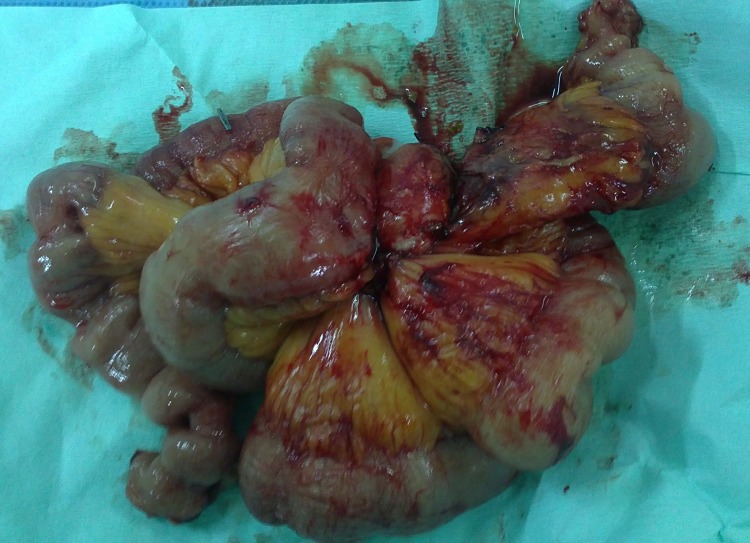
Resected mesenteric mass

On laparotomy, a 5cm × 5cm mass was found in the mesentery of the ileum and multiple small liver metastases in both liver lobes were observed. The mesenteric mass and adjacent small bowel were resected and a bilateral adrenalectomy was performed (Fig 3). The multiple liver metastases in both liver lobes were not amenable to a metastasectomy and just one of them was biopsied.

The pathology examination revealed adrenal cortical hyperplasia in both resected adrenals, a well differentiated neuroendocrine carcinoma in the resected mesenteric mass, no tumoural involvement in four resected lymph nodes and metastasis of neuroendocrine carcinoma in the liver lesion. The immunohistochemistry examination on the mesenteric mass was positive for chromogranin A, synaptophysin and cytokeratin, and negative for epithelial membrane antigen, confirming the diagnosis of NET.

The patient had an uneventful post-operative period and was referred to the endocrinology ward for adrenal hormone replacement therapy. ACTH and cortisol levels were both reduced after surgery. At the one-year follow-up appointment, she had been in a good general condition with no significant problems. Her follow-up colonoscopy and chest CT were normal. The abdominal CT did not reveal any lesions other than her previous liver metastases, which were stable in size.

## Discussion

Ectopic Cushing’s syndrome results from inappropriately high levels of ACTH, secreted by various types of tumours. These tumours consist of NETs, islet cell tumours, small cell lung carcinomas, medullary thyroid cancers, pheochromocytomas, carcinomas of the thymus and pancreas tumours.^1–4^

NETs originate from neuroendocrine cells, which are widely distributed throughout the body. They secrete various substances and hormones including ACTH. These substances result in diverse clinical presentations. NETs most commonly involve the lungs and gastrointestinal system. They have also been reported in other sites such as the ovaries, prostate, lymph nodes and cervix.^1,5–7^ Gastrointestinal NETs usually involve the small bowel, rectum, appendix and pancreas. Primary mesenteric NETs are extremely rare and very few cases of primary mesenteric involvement have been reported worldwide.^1,7,8^

The clinical presentation of these tumours depends on their location, and the types of hormones and substances they secrete. NETs that produce ACTH can result in ectopic Cushing’s syndrome. In these patients, serum ACTH levels are high and not suppressed in response to a high dose of dexamethasone (dexamethasone suppression test). In ectopic ACTH secretion, an octreotide scan and CT are very helpful in localising the lesions. In the majority of cases, there would be no need for further diagnostic workup although they may be missed if not searched for meticulously.^1^

Other diagnostic modalities such as radioguided surgery have been proven helpful in a number of cases.^1,4^ In radioguided surgery, radiolabelled pentetreotide is injected pre-operatively and positive sites are detected using a gamma counter intra-operatively. In our case, the use of CT and an octreotide scan was sufficient for localising the tumour site.

To make the diagnosis of mesenteric NET, one must first rule out other primary sites by the use of CT, colonoscopy, small bowel series and scintigraphy.^8^ In our patient, abdominal CT and an octreotide scan exhibited a mesenteric tumour and liver metastases. A colonoscopy, small bowel series and surgical exploration of the abdomen can confirm the diagnosis of mesenteric NET.

NETs have specific immunohistochemistry features. Synaptophysin, chromogranin A, cytokeratins and neuron-specific enolase are usually positive.^1,7,8^ In our case, synaptophysin, chromogranin A and cytokeratin were positive, which confirmed the diagnosis of NET.

Medical and surgical methods can be used for the management of hypercortisolism and its complications. Medications such as metyrapone and aminoglutethimide are somewhat effective in symptomatic control but surgical interventions such as a bilateral adrenalectomy are preferred in patients who do not respond to medical treatment.^2^ As for our patient, although the tumour was resectable, due to ACTH secretion by liver metastases, a bilateral adrenalectomy with subsequent hormone replacement therapy was the treatment of choice. It should be noted that resection of NETs is recommended despite the presence of liver metastases as cytoreduction improves symptoms, provides palliation and can increase survival.^9^

## Conclusions

Primary mesenteric involvement in NETs is extremely rare and very few cases have been reported worldwide. After a thorough investigation and ruling out other possible primary sites, it should be kept in mind that the mesentery can also be a primary site for NETs, causing similar symptoms and requiring similar treatment options.

## References

[1] Fasshauer M, Lincke T, Witzigmann H*et al.* Ectopic Cushing syndrome caused by a neuroendocrine carcinoma of the mesentery. BMC Cancer 2006; 6: 108 16643652 10.1186/1471-2407-6-108 PMC1464147

[2] Aniszewski JP, Young WF, Thompson GB*et al.* Cushing syndrome due to ectopic adrenocorticotropic hormone secretion. World J Surg 2001; 25: 934–940 11572035 10.1007/s00268-001-0032-5

[3] Uecker JM, Janzow MT. A case of Cushing syndrome secondary to ectopic adrenocorticotropic hormone producing carcinoid of the duodenum. Am Surg 2005; 71: 445–446 15986979

[4] Grossrubatscher E, Vignati F, Dalino P*et al.* Use of radioguided surgery with [111In]-pentetreotide in the management of an ACTH-secreting bronchial carcinoid causing ectopic Cushing’s syndrome. J Endocrinol Invest 2005; 28: 72–78 15816375 10.1007/BF03345533

[5] Koch CA, Azumi N, Furlong MA*et al.* Carcinoid syndrome caused by an atypical carcinoid of the uterine cervix. J Clin Endocrinol Metab 1999; 84: 4,209–4,213 10.1210/jcem.84.11.6126 10566674

[6] Orbetzova M, Andreeva M, Zacharieva S*et al.* Ectopic ACTH-syndrome due to ovarian carcinoma. Exp Clin Endocrinol Diabetes 1997; 105: 363–365 9439934 10.1055/s-0029-1211780

[7] Kitchens WH, Elias N, Blaszkowsky LS*et al.* Partial abdominal evisceration and intestinal autotransplantation to resect a mesenteric carcinoid tumor. World J Surg Oncol 2011; 9: 11 21281518 10.1186/1477-7819-9-11 PMC3038967

[8] Kimchi NA, Rivkin G, Wiener Y*et al.* Primary neuroendocrine tumor (carcinoid) of the mesocolon. Isr Med Assoc J 2001; 3: 288–289 11344846

[9] Chambers AJ, Pasieka JL, Dixon E, Rorstad O. The palliative benefit of aggressive surgical intervention for both hepatic and mesenteric metastases from neuroendocrine tumors. Surgery 2008; 144: 645–651 18847650 10.1016/j.surg.2008.06.008

